# Perceptions of overweight by primary carers (mothers/grandmothers) of under five and elementary school-aged children in Bandung, Indonesia: a qualitative study

**DOI:** 10.1186/s12966-017-0556-1

**Published:** 2017-07-27

**Authors:** Cut Novianti Rachmi, Cynthia Louise Hunter, Mu Li, Louise Alison Baur

**Affiliations:** 1 Discipline of Child & Adolescent Health, The Children’s Hospital at Westmead, University of Sydney Clinical School, Locked Bag 4001, Westmead, NSW 2145 Australia; 2 School of Social and Political Sciences, The University of Sydney, RC Mills Building (A26), Sydney, NSW 2006 Australia; 3 Sydney School of Public Health, The University of Sydney, Edward Ford Building (A27), Fisher Rd, Sydney, NSW 2006 Australia

**Keywords:** Overweight, Children, Perceptions, Mothers, Indonesia, Qualitative

## Abstract

**Background:**

The prevalence of childhood overweight has increased in the past two decades in Indonesia. Even though prevalence is escalating, there is a lack of qualitative evidence to assist in the design and implementation of strategies to tackle this issue. This study aimed to explore the view of primary carers (mothers and grandmothers) from different socio-economic-status groups, on childhood overweight in the Greater Bandung Area, Indonesia.

**Methods:**

We conducted 12 focus groups discussions with a total of 94 carers of under-five and 7–12 years children, from June to October 2016. We used the grounded theory approach in our analysis.

**Results:**

Three main categories emerged: the concept of overweight, factors contributing to overweight, and awareness and feelings towards overweight children. Most carers from all SES groups defined overweight subjectively, while a few from the low SES group defined it objectively. Most carers from low and high SES groups agreed with the concept “chubbier is healthier”. All carers had some knowledge of the main factors that contribute to childhood overweight: dietary factors, activity levels and sedentary behavior, and hereditary factors. Carers from all SES groups described similar characteristics of overweight; carers from low and intermediate SES groups had mixed feelings while all high SES carers have negative feelings about overweight children, mostly related to stigma. However, carers who identified their own children as being overweight expressed sensitivity about this weight status, especially their physical abilities. Almost all carers knew their children’s current weight while less than two thirds knew their children’s height.

**Conclusions:**

There are several policy implications. Firstly, health-related knowledge of the primary carers is of great importance and needs augmenting. To increase that knowledge, there is a role for front-line health practitioners (doctors/midwives/nurses) to be more active in educating the community. Secondly, simpler and more effective ways to disseminate healthy lifestyle messages to carers is required. Thirdly, by placing more emphasis on carers monitoring their children’s growth may encourage carers to take steps to keep their children in the healthy weight and height ranges. Fourthly, the Department of Education may need to improve the quality and quantity of physical activity in schools.

**Electronic supplementary material:**

The online version of this article (doi:10.1186/s12966-017-0556-1) contains supplementary material, which is available to authorized users.

## Background

Overweight/ obesity in both children and adults, while initially an issue mainly in western countries, is now a major emerging health problem in countries undergoing economic transition – including Indonesia [1, 2]. Indonesia, the world’s fourth most populous country, has been dealing with the problem of children’s undernutrition, including stunting and underweight for decades, as a consequence of which many nutrition policies and programs were focused on addressing these issues [3]. Overweight, on the other hand, has only been recognised as an Indonesian health problem for little more than a decade [2, 3].

The prevalence of childhood overweight in Indonesia has significantly increased in the last 20 years, with higher prevalence rates found among boys in general and boys and girls living in urban areas [2]. To our knowledge, qualitative evidence about overweight in Indonesia is scarce, with the available evidence mainly focused on mothers with overweight children, [4] and none considering the views of people from different socio-economic groups.

Parents, particularly mothers, hold an important role in child feeding, role modelling for healthy eating and active living, and the prevention of overweight [5–11]. Many potential strategies in tackling overweight will involve working with families, especially the primary carers of young children. These strategies will benefit from an understanding of the knowledge, attitudes, and behaviours of the primary carers. Thus, this study aimed to explore the perceptions of Indonesian mothers and grandmothers from different socio-economic groups towards child overweight/ obesity and related issues. In the Indonesian language, people refer to overweight/ obesity with the same word, *kegemukan*, except those in the health sector/ academics. Hereafter, we will refer to overweight/ obesity as overweight.

## Methods

### Study design and location

We undertook a qualitative study in Bandung, West Java, Indonesia. West Java Province consists of 27 cities/regencies and we selected three cities/regencies (Bandung City, Bandung Regency, and Bandung Barat Regency) based on high population numbers and mixed ethnic groups, to explore the situation in Indonesia [12]. Every district in each city/regency was numbered and we chose the selected areas based on the last digit from a table of random numbers. These three areas have different Human Development Index scores (high, medium, and low); these were used as a measure of different levels of socioeconomic status [12]. The locations of the selected areas (Cisarua, Margahayu, and Sukasari districts) represent the low, intermediate, and high socio-economic status areas, respectively.

The rationale for selecting areas of different SES was to obtain a diversity of views of mothers/grandmothers towards children’s growth, development and overweight and to observe whether SES is a contributing factor to overweight. Hereafter, we will refer to mothers/grandmothers as carers.

### Participants and recruitment process

**Fig. 1:**
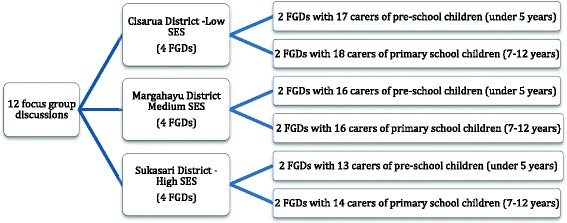
Numbers and participants of focus group discussions in Bandung, Indonesia. SES: socio economic status; Low SES: FGDs 5, 6, 7, and 8; Intermediate SES: FGDs 3, 4, 7, and 8; High SES: FGDs 1, 2, 11, and 12

Twelve focus group discussions (FGDs) were conducted, six with carers of under five children and six with carers of elementary-school-aged children (7–12 years),^1^ from June to October 2016 (Fig. 1). Inclusion criteria were carers of children who hold or share the primary role in decision-making for the care of the child or were responsible, in part or in whole, for child feeding and eating, including food shopping and budgeting.

To recruit participants from low and intermediate SES areas, we contacted Community Health Centres (*Puskesmas*) in the chosen areas to reach carers of under five children. We collaborated with local midwives and health *kader* (mothers who are trained and work for the *Puskesmas* and Community Health Posts [*Posyandu*] for a small incentive). These *kader* have knowledge of the people living in the communities [13]. We provided a verbal explanation of the project, and disseminated the participant information statement to invite participation. To recruit carers of 7–12 years children, we asked for assistance from class coordinators in public elementary schools (a mother from each grade in elementary school who acts as the information source for fellow mothers). We also placed project information including contact details in students’ communication books (book that teachers use to communicate with mothers of individual children).

Recruitment of participants from high SES groups was by invitation to local private schools and paediatricians’ offices. In Indonesia, children from socially advantaged families do not go to *Puskesmas*, but to private medical practices or specialists, and they attend private schools. We used the same approach in each school (i.e. contacting the class coordinator), and asked the nurse in the paediatrician’s office to assist us in providing information about and contact details for the study to the carers.

Interested carers then directly contacted the research team who gave further explanation about the discussion process and took details. Carers who gave verbal consent to participate were later approached to set the date and time for the FGD.

### Research team, data collection and approach to analysis

The field research team consisted of the first author (CNR) and two research assistants (DMP and TMF). They underwent a three-day training program in qualitative research, concentrating on FGDs, with CLH in Bandung. Question guidelines were developed in English and finalised during the training. Afterwards, the guidelines were translated into the Indonesian and Sundanese languages and a pilot FGD was conducted to test the questions guidelines (Table 1). This process included a discussion of potential issues and possible resolutions that might be encountered during the FGDs.

**Table 1:** Questions used in focus group discussions in Greater Bandung Area, Indonesia

No	Questions (English)	Indonesian	Sundanese
1	There is quite a lot of talk around these days about being obese or being overweight in both adults and in children and babies - can you tell me what you think this means? When I say overweight/obesity, what is the first thing that comes in your mind? In your opinion, which celebrities have the ideal body?	*Akhir-akhir ini banyak pembicaraan tentang terlalu gemuk pada anak dan dewasa, termasuk bayi, menurut ibu, apa arti terlalu gemuk? Ketika saya mengatakan terlalu gemuk, apa hal pertama yang ibu pikirkan? Menurut ibu, artis yang berat badannnya ideal siapa?*	*Jaman ayeuna seuer pisan pacariosan tentang bayi, barudak sareng dewasa anu parawakanna ageung teuing, menurut ibu, kumaha artina ari parawakanna ageung teuing teh? Upami abdi nyarios parawakan anu ageung teuing, naon hal pertama anu ibu pikirkeun? Menurut ibu, saha artis anu parawakanna ideal?*
2	In your opinion, when can you say a baby/child is overweight/obese? Prompts: e.g. they have multiple chins, they wear clothes several sizes bigger than their age, they have trouble learning how to walk/run, they eat adult size portion of food	*Menurut ibu, kapan seorang bayi/anak bisa dibilang terlalu gemuk?* *Bantuan:* • *Mukanya (dagu berlipat)* • *Ukuran baju* • *Terlambat belajar berjalan/lari (bayi)* • *Makan banyak seperti orang dewasa*	*Menurut ibu, iraha bayi/barudak teh disebat parawakanna ageung teuing?* *Bantuan:* • *Rarayna (dagu berlipat)* • *Ukuran bajuna* • *Telat diajar jalan/lumpat(bayi)* • *Tuangna siga jalmi dewasa*
3	What do you think of the saying ‘chubbier is healthier’? What do you think about the relationship between body weight and health?	*Apa pendapat ibu tentang ungkapan “lebih gemuk lebih sehat”? Bagaimana pendapat ibu tentang hubungan berat badan dengan kesehatan?*	*Kumaha pendapat ibu tentang paribasa “langkung ageung langkung sehat”? Kumaha pendapat ibu tentang hubungan abot badan sareng kasihatan?*
4	What factors do you think contribute/ influence to a baby/ child’s weight? Prompts: Is it dietary? Mention the following only if they are not mentioned: genetic, family eating patterns, physical activity, screen time (length of time spends in front of a screen – TV, computers, iPad, video games, reading, phone/games, playing cards, board games, etc), sleep, other activities (soccer, kite flying, hide and seek, hop scotch, jump rope, other outside activities: buying something at store)? In your knowledge, what do your children usually have as snacks? Prompts: • Soda • Instant noodles How do you feel about your children’s snacks choices?	*Menurut ibu, hal apa saja yang mempengaruhi berat badan bayi/anak?* *Bantuan: Apakah makanan?* *Sebut hal berikut hanya bila hal-hal ini tidak disinggung: turunan/ bawaan (genetik), pola makan keluarga, kegiatan fisik, waktu layar (waktu yang dihabiskan di depan layar - TV, komputer, iPad, permainan video, membaca, telefon/ permainan, bermain kartu, permainan lainnya), tidur, kegiatan lainnya (main bola, layangan, petak umpet, engkle, lompat tali, kegiatan lain di luar rumah: belanja ke warung)?* *Sepengetahuan ibu, apa yang dimakan anak ibu sebagai cemilan?* *Bantuan:* • *Soda* • *Mie instan* *Bagaimana perasaan ibu tentang pilihan cemilan anak ibu?*	*Menurut ibu, naon wae anu ngaruh kana berat badan bayi/anak?* *Bantuan: Apakah makanan?* *Sebut hal berikut hanya bila hal-hal ini tidak disinggung: turunan/ bawaan (genetik), pola makan keluarga, kegiatan fisik, waktu layar (waktu yang dihabiskan di depan layar - TV, komputer, iPad, permainan video, membaca, telefon/ permainan, bermain kartu, permainan lainnya), tidur, kegiatan lainnya (main bola, layangan, petak umpet, engkle, lompat tali, kegiatan lain di luar rumah: belanja ke warung)?* *Sapengetahuan ibu, naon wae cemilan anu dituang ku anak ibu?* *Bantuan:* • *Soda* • *Mie instan* *Kumaha perasaan ibu tentang pilihan cemilan anak ibu?*
5	How much do you expect your baby to weigh at birth? About how much do you think a healthy baby should weigh by 6 months and by age 12 months?	*Menurut ibu berapa berat badan bayi yang diharapkan saat lahir? Menurut ibu berapa seharusnya berat badan bayi saat usia 6 bulan dan 1 tahun?*	*Menurut ibu, sabaraha abot bayi anu diharapkeun pas lahir? Menurut ibu, sabaraha kedahna abot bayi pas usia 6 sasih sareng sataun?*
6	Can you tell me how much your child weighs and his/her height now?	*Apakah ibu tahu berapa berat badan dan tinggi badan anak ibu sekarang?*	*Kinten-kintena ibu terang teu sabaraha abot sareng tinggi anak ibu ayeuna?*
7	Are you satisfied with your child’s growth up to this point? If there is anything you would like to change about your child’s weight, what would it be?	*Apakah ibu puas dengan pertumbuhan anak ibu sampai saat ini? Kalau ada hal yang ingin diubah mengenai berat badan anak ibu, kira-kira hal apa?*	*Apa ibu puas sareng pertumbuhan anak ibu dugika ayeuna? Upaya aya hal anu bade diubah soal berat badan anak ibu, naon anu bade diubah?*
8	Who do you think plays an important role in influencing your child’s weight in the house? Prompts: • Who is responsible in determining the kind of food the child consume? • Who feed the child?	*Ibu, kalau di rumah siapa yang berperan dalam pertumbuhan anak ibu?* *Bantuan:* • *Siapa yang menentukan jenis makanan anak?* • *Siapa yang memberikan makanan anak?*	*Ibu, upami di bumi saha anu berperan dina pertumbuhan anak ibu?* *Bantuan:* • *Saha anu nangtoskeun jenis makanan anak?* • *Saha anu masihan makanan anak?*
9	Have you ever had any discussions regarding your baby/ child’s growth? If yes, what is the discussion about? Prompts: For example if you think he/ she is the tallest/ biggest one in the class/ neighbourhood? With who do you usually have the discussion with? Prompts: • Husband • In laws • Posyandu (health post) *kader* • *Doctor* • *Neighbours*	*Apakah ibu pernah berdiskusi tentang pertumbuhan anak ibu?* *Mengenai apa?* *Bantuan: Misalnya apakah menurut ibu anak ibu yang paling tinggi/ besar di kelas/ lingkungan.* *Dengan siapa?* *Bantuan:* • *Suami* • *Mertua* • *Kader posyandu* • *Dokter* • *Tetangga*	*Apa ibu pernah diskusi tentang pertumbuhan anak ibu?* *Diskusina tentang apa?* *Bantuan: Misalnya apa menurut ibu anak ibu nu pang tinggi/ besarna di kelas/ lingkunganna.* *Sareng saha?* *Bantuan:* • *Suami* • *Mertua* • *Kader* • *Dokter* • *Tatanggi*
10	What do you think when you see children who appear overweight/obese? Can you elaborate more on that?	*Apa yang ibu pikirkan ketika melihat anak yang menurut ibu terlalu gemuk? Tolong jelaskan!*	*Naon anu ibu pikirkeun upami ningali anak anu menurut ibu parawakanna ageung teuing? Tiasa dijelaskeun bu.!*

CNR acted as a moderator in all of the FGDs, with two research assistants (DMP and TMF) observing and managing the recordings and time keeping. Prior to each FGD, the study team explained the FGD process to the participants and asked them to fill in the basic demographic information form and provide written informed consent. Participants were offered the language of their choice to be used during the FGDs, either Indonesian or Sundanese (the language of West Java), in which all three moderators were fluent. Each FGD began with a 30-min ice-breaker, to build rapport between participants and researchers. We highlighted how much everyone’s opinion was valued and encouraged the participants to speak freely. Similarly, and to ensure confidentiality, we did not videotape the discussion. To ensure consistency of the discussion the moderator used the same guideline, including a set of prompt questions. All discussions were digitally recorded, and lasted 50–90 min. Group size ranged from 6 to 9 people. Participants each received 11 USD for participation.

Transcription and translation started immediately after the first FGD, giving the team some time to reflect on the discussion results. Transcription was done only in Indonesian by DMP and TMF and checked by CNR to confirm that the transcripts were verbatim to the recordings. The research assistants then translated the transcripts into English; both Indonesian and English transcripts were checked by CLH and CNR to ensure the quality of the translation. Sundanese or Indonesian terms that have no exact English translation were kept and explained in English inside parentheses next to those terms.

We used the grounded theory approach in our analysis [14–16]. All transcripts were analysed using NVivo for Mac version 11.0.0 [17]. The research assistants were not involved in the analysis phase. The analysis was conducted following the steps: 1) CNR and CLH read all transcripts several times to familiarise themselves with the data. 2) CNR undertook the open coding and compared the coding within and between transcripts. 3) CNR and CLH identified categories and sub-categories of ideas/concepts. 4) Once the final version of categories and subcategories was agreed upon, a final combination of subcategories took place. 5) CNR and CLH identified similarities and differences between and within the different SES groups. 6) The quotations that best represented each category and subcategory were identified. As part of the analysis, CNR quantified comments throughout the transcripts looking at responses from individual carers. Quotations representing each SES grouping were selected from multiple FGD groups. 7) All authors (CNR, CLH, ML, and LAB) agreed upon the final version of the categories and subcategories. LAB and ML, experts in childhood obesity and childhood nutrition, were significantly involved in the design phase of the research and writing up of the manuscript.

### Credibility and trustworthiness

Credibility and trustworthiness were addressed in several ways. One researcher (CNR) was the moderator of all 12 FGDs and could, therefore, describe the situation and convey the opinions stated directly (words) or indirectly (intonation of voice or facial expressions) consistently. We reduced interpretive bias by involving another analyst (CLH) who was not present during the data collection. CNR also conducted triangulation with the two research assistants and used the notes made during the FGDs to ensure the nuances captured during the discussions were agreed upon by three facilitators.

## Results

### Characteristics of participants

A total of 94 carers gave their consent and participated in the FGDs. We had similar numbers of carers in the under 5 years and elementary groups. Most carers from the low and intermediate SES groups had graduated either from elementary or middle and high school, but none from university. In contrast, most carers from the high SES groups were university graduates with only a few having middle and high school qualifications alone. We had more mothers compared to grandmothers in all three SES groups (Table 2). We had carers from 11 different ethnicities; however, we did not find that ethnicity played a part in the differences in the discussions.

**Table 2:** Characteristics of participants from 12 focus group discussions in Greater Bandung Area, Indonesia

	Low SES (groups 5, 6, 7, 8)	Intermediate SES (groups 3, 4, 9, 10)	High SES (groups 1, 2, 11, 12)
Number of participants			
Total	35	32	27
Under five	17 (48.6%)	16 (50.0%)	13 (48.1%)
Elementary	18 (51.4%)	16 (50.0%)	14 (51.9%)
Education			
Elementary or less	16 (45.7%)	11 (34.4%)	0
Middle & High School	16 (45.7%)	19 (59.4%)	3 (11.1%)
Diploma	3 (8.6%)	2 (6.2%)	6 (22.2%)
University or higher	0	0	18 (66.7%)
Distribution of participants			
Mothers	31	23 (71.9%)	25 (92.6%)
Grandmothers	4	9 (28.1%)	2 (7.4%)
Number of children at home			
Two or less	24 (68.6%)	16 (50.0%)	17 (63.0%)
More than two	11 (31.4%)	16 (50.0%)	10 (37.0%)
Ethnicities	5 (Sunda, Jawa, Padang, Batak, Bali)	5 (Sunda, Jawa, Betawi, Minahasa, Aceh)	7 (Sunda, Jawa, Riau, Dayak, Batak, Betawi, Bugis)

*SES* socio economic status

### Emerging categories

Emerging categories are shown in Table 3. There were several marked differences between the three SES groups involved. Although we expected differences would arise from carers of children under 5 years old and carers of elementary school children within the same SES group, such differences were not found.

**Table 3:** Category and subcategories on the perception of overweight in carers in Greater Bandung Area, Indonesia

Category	Subcategory
1. Concept of overweight	1.1 How carers define overweight
	1.2 Normal weight range
	1.3 Chubbier is healthier
	1.4 Ideal body shape
2. Factors contributing to overweight	2.1 Dietary factors
	2.2 Activity levels and sedentary behaviour in children
	2.3 Heredity
3. Awareness and feelings towards overweight in children	3.1 Characteristics of overweight
	3.2 Food portion size in overweight children
	3.3 Eating frequency in overweight children
	3.4 Feelings about overweight children
	3.5 Sensitivity in carers of own overweight children
	3.6 Children’s current weight and height

### Concepts of overweight

This broad category portrays carers’ opinions around the concepts of overweight in children, “chubbier is healthier” and the ideal adult body, including their expectations of normal weight ranges at certain ages. Relevant excerpts from the transcripts are presented in Table 4.

**Table 4:** Quotes on concept of overweight according to carers from Bandung, Indonesia

	Low SES (FGD 5, 6, 7, 8)	Intermediate SES (FGD 3, 4, 7, 8)	High SES (FGD 1, 2, 11, 12)
How carers define overweight	• P1: Too much fat. • P3: Big body. • P4: Posture not appropriate with height and age range. • P8: Weight exceeds normal range based on age, based on the chart in KMS-Kartu Menuju Sehat (Health Card)	• P2: Excessive fat. • P5: Body weighs more than it should be. • P7: Around here it means you are healthy. • P6: In here it means you are happy.	• P1: Weight and height not balanced/ proportional. • P4: (Someone who has a) big body. • P8: Excessive weight. • P7: Obesity is the same with overweight. • P8: Weight is more than standard; I know there’s a way to measure it.
Normal weight range	• Birth: 2.5–3.5 kg • 6 months: 5–9 kg • 1 year: 9–12 kg	• Birth: 2.8–3.5 kg • 6 months: 6–8 kg • 1 year: 7–11 kg	• Birth: 2.5–3.5 kg • 6 months: 8–10 kg • 1 year: 8–13 kg
Chubbier is healthier	• P5: I personally don’t know what measurements would count as obese, what obesity is. But according to my knowledge... I agree with “the chubbier, the healthier”. Being chubby is healthy. That’s what I think. • P4: But, if it’s me, Doc, I want to have a fat child. Who doesn’t want a fat child, right? Fat and healthy, they’re so nice to look at. • 1P2: If you mean a healthy fat, well... then that is okay, maybe. But... if it’s unhealthy fat, it means they have too much. • P5: Cholesterols. • P7: I want a child who is fat and healthy.. it would be better.. uh, I want it to be that way.	• P4: I think it’s cute, but definitely not healthier. • P8: (They have) lots of diseases. • P3: If they’re too fat, they are often sick and that’s not good. • P1: Well, there are fat people who are healthy and unhealthy. • P7: I think I agree with chubbier is healthier, if it’s in toddlers. And also in babies, I think	• P3: Umm… When she was 6 months old, the doctor said she was obese. So yeah, she was fat, chubby. At the beginning I thought she was cute. But when the doctor said she was obese, it frightened me. • P2: My child often got an asthma attack when she was fat. • P6: It’s (chubbier is healthier) not true. • P7: The child can be big, but actually, the child is not healthy.
Ideal body shape	• P6: Luna Maya (famous model and movie star in Indonesia). • P7: Krisdayanti (famous singer in Indonesia), she still has a nice body even though she has children. • P8: Ideally, approximately 40 kg. • P3: Being ideal is about having nice height, too. P2:Steven William (half Indonesian, half English movie star). • P6: Like a model, hahaha.		

#### How carers define overweight

Most (23 out of 27) carers in the high SES category defined overweight subjectively, for example through visual interpretation of body size. The researchers did not provide any particular definition of overweight nor differentiate between overweight and obesity at the beginning of our discussions, since most Indonesians use the same word, *kegemukan*. One carer stated that in her opinion the two terms are the same. Few (4/27) high SES carers defined it in a more objective way (i.e. based on specific measurements).

In contrast, although the same subjective pattern was seen in the low SES groups (32/35), three carers defined overweight objectively, based on Kartu Menuju Sehat (KMS – Health Card). KMS is a card used since the 1970s in Indonesia (Additional file 1). In 2010, the Indonesian Ministry of Health released a regulation to use this card for children aged under 5. The purpose of the KMS is to monitor children’s growth, to record children’s immunisation history, health services usage, and a tool to educate the community [18].

All carers (32/32) from the intermediate SES groups defined overweight in a subjective way. A few (3/32) described overweight differently.

#### Normal birthweight range

Carers from different SES groups had similar expectations for a baby’s birthweight. Carers from high SES groups expected their children to be heavier at age 6 months and 1 year, compared to the other two groups.

#### “chubbier is healthier”

One third (12/35) of carers from the low SES groups agreed and two thirds (23/35) disagreed with the term “chubbier is healthier”. They mentioned the concept of healthy overweight as an overweight baby/child who is still very active and is developmentally normal.

All carers (32/32) from the intermediate SES groups were against the concept of “chubbier is healthier”. They specifically differentiated between cuteness and health status in overweight children. However, they also included the concept of healthy overweight.

One fourth (7/27) of the high SES carers agreed with “chubbier is healthier”, while the rest (20/27) disagreed, highlighting past experiences of dealing with the overweight status of their children.

#### Ideal body

Carers from all three SES groups referred to Indonesian media celebrities they considered had ideal bodies. The ideal body was described as tall, slim, skinny, or model-like in women and a well-muscled body in men. However, it was difficult to name child celebrities they felt had an ideal body.

### Factors contributing to overweight

This category represents carers’ views on what they considered were the three main factors contributing to overweight: dietary factors, physical activity and sedentary behaviours, and heredity (Table 5).

**Table 5:** Quotes on factors contributing to weight according to carers from Bandung, Indonesia

	Low SES	Intermediate SES	High SES
Dietary factors	• P1: It’s mainly caused by one’s dietary habit that is too... where one does not have appropriate portions. Also, frequent eating can cause obesity. • P4: It could be that, Ma’am, aa, obesity comes from eating too many sugary foods, excessive sugar consumption. Or it could be from the snacks, Ma’am; they’re not very healthy. • P7: Even if he has eaten two packs of noodles, if he has not eaten rice, he’ll consider it as if he hasn’t eaten yet.	• P2: They eat too much P5: It has to be empat sehat lima sempurna (Four Healthy, Five Perfect – an Indonesian ideal meal incorporating carbohydrates, proteins, vegetables, fruits, and milk). • P2: Like when you sleep right after you eat, lack of exercise, eating snacks… these things make you bigger. • P9: Aa, they eat a bowl of noodle and a plateful of rice. M: So, noodles and rice. How often do they eat that, Ma’am? P9: Aa, sometimes they eat those twice a day. P5: Noodle can also cause obesity. • P7: Initially, A (name) drank too many sugary drinks like... something like Teh Gelas (sugary drinks brand). So he became obese.	• P1: We are Indonesians, we have to eat rice. • P7: She has a big appetite, drinks a lot of milk, and her father is also fat. Could that be a factor? • P2: I rarely consume noodles now, when I do, I only put half of the MSG. Hahaha. • P6: So you add salt to it? • P2: Aa, nope, I add eggs and mix them with butter, so it has the right amount of saltiness from the butter. • P6: But that’s where the fat come from (the butter). • P4: It can be the quantity of the milk.P5: It can be how much the children drink the milk. • P3: Maybe it’s because they drink a lot of milk. • P4: For example, he drinks brand A, although he only drinks a little, but the composition is different. Maybe one of the compositions stimulates the appetite.
Activity levels and sedentary behaviours in children	• P1: After they eat, they’re just sitting down, watching TV. So that, aa, there is no chance for them to burn the calories. That might have caused (the person to be). • P7: Fat. • P2: He is out somewhere to play (right after he came home from school). Changes his uniform, then goes straight outside to play. • P3: After school my daughter usually have her lunch, then….. Then after lunch, that’s in... in the afternoon, aa, she is joining Qur’an (Moslem’s holy book) recitation. • P7: Maybe, being fat is caused by lack of...maybe lack of activities? • P8: They fly kites. • P5: They just run around. • P8: They ride bicycles, Doc. • P7: Right, bicycles.	• P6: Lifestyle. M: What kind of lifestyle? P6: One that lacks exercise. • P8: My child can’t stand staying at home, so he plays outside. • M: What do you think about the physical education class at school? • P6: It’s not long enough. • P4: Right, it’s not enough. • P3: They mostly play outside, of course.P5: Yeah, outdoors. • P8: He reads Qur’an in the evening. • P5: He reads the Qur’an, ma’am. • P3: My child reads the Qur’an in the evening and afterwards he plays for a while M: Outside or inside the house? • P3: He plays outside.	• P4: Video games. • P5: This (imitates someone playing video games) isn’t being active. • All: Hahaha • P4: Because, the children can, umm, even if they don’t go outside, they can do the activities at home.P3: They can play around the house, moving here and there, right? P4: Yes. They can go up and down. P5: On the stairs, right? • P2: For my son of course it’s inside. • P3: Heeh, he arrived in the afternoon, and then go straight to his math course. And then it’s Maghrib (praying times-6 pm) when he came home; he only spends some time outside the house on Saturdays and Sundays, and that doesn’t happen a lot either. • P2: Yeah, same here. Even on Saturdays and Sundays he doesn’t spend much time outside.
Heredity	• P1: Those who have inherited it from.... • P6: From the parents. • P3: Being large is in my family’s blood • P8: But he inherits it from his father. His father is also fat. • P2: That’s when an excessive diet is not balanced by exercise, which was mentioned earlier. Heredity is a factor that can play into this, too, right? Like genes? Right? • P9: People in the village always think like this, it’s normal if the children are fat because the father is also fat, just like that.	• P8: There’s the heredity factor. • P3: Yes, it’s inherited. • P5: That’s why the children become fat. • P7: If the parents were fat, the children would also be fat. • P1: Heredity. • P2: It can be inherited. • P4: Fatness can be inherited.	• P2: It’s inherited. I am one of the big families. Yeah, my husband and I, we’re big and tall people. • P7: Several diseases are heredity like cancer, asthma, but not overweight. • P8: The shape and size of the body is probably not affected by heredity. • P6: It might be both. Maybe, the fat people already has a heredity factor and then… • P5: It’s worsened by wrong dietary habit. • P6: The heredity, maybe, heredity factor? • P4: Do you mean genetic factor? • P6: So, their bones are naturally big. • P5: Yes, since they were little, it’s already big.

#### Dietary factors

Carers from all SES groups stated that there was a range of dietary factors contributing to overweight such as excessive sugar consumption, unhealthy snacks, certain types of food, big portions, frequent eating, and parental eating/snacking habits.

Intermediate and high SES groups commented upon vitamin supplements, milk, cooking oil, and mealtimes (especially dinner). Diets high in carbohydrate (rice or noodles) were also a factor.

Rice is Indonesia’s staple food. All SES groups considered rice as a “must have”, everyday food. One mother (high SES group) admitted that it is part of their identity as an Indonesian.

It seems that what the family will cook/eat depended heavily on what the children requested, because if the carers did not fulfill the children’s wishes the children’s behaviour may become difficult, for example by being cranky or do not want to eat anything through out the day. Most carers (25/32) from intermediate SES groups admitted that their children were heavily influenced by TV food commercials.

Low and intermediate SES carers in particular mentioned that the ideal menu should consist of *empat sehat lima sempurna* (Four Healthy, Five Perfect – an Indonesian ideal meal incorporating carbohydrates, proteins, vegetables, fruits, and milk). This concept, introduced by the Indonesian government in 1952, is now considered dated because it only gives information about variety of food without considering quantity [19]. In 1995 the government introduced new guidelines *Pedoman Umum Gizi Seimbang* (PUGS - Balanced Nutrition Guidelines) that contain information on both food variety and portion size along with the importance of exercise and water consumption [19, 20]. None of the carers mentioned PUGS.

#### Activity levels and sedentary behaviours in children

All elementary schools in Indonesia have a mandatory 2 h physical education class per week. This was considered not enough by all low and intermediate SES carers, who said children should engage in more physical activity. Carers stated that boys spent their time in PE class by playing soccer and girls by having aerobic activities. They also mentioned the 2 h includes time to change clothes and sometimes time to walk to the nearby sports field (low and intermediate SES), leaving only 45–60 min for playing sports. All children in the low SES groups went to public schools (free of any charge), while children in intermediate SES groups went to public schools or Islamic schools (madrasah; some fee payment required but not as expensive as private schools). Public kindergartens start at 7.30 am and end at 9.30 am, 1st-3rd grades of elementary schools start at 7.00 am and end at 10.00 am, while 4th–6th grades start at 7.00 am and end at 12.00 pm. Children from low and intermediate SES groups spent their time after school playing outside although they also had screen time (watching TV at night). These families usually had “physical activity time” once a week such as hiking the mountains, running, and family sports time. In many low and intermediate SES areas in Indonesia children must spend time (from 5 pm–6.30 pm) in Quran recitation class every day at the mosque.

In contrast, children from high SES groups spent most of their after school time indoors at home. In addition to the school physical education class, there were extracurricular activities such as dancing, martial arts, and soccer. Eight (8/27) carers thought that, ideally, children should have more (maybe around 3 h) physical activities outside school every day because the activities at school are not enough. Half (13/27) of the carers stated children do not need extra activity outside school because they are moving around the house anyway - they have bigger houses. All children in these groups went to private schools. Classes in private schools start at 7 am and finish at 11.30 am for kindergarten, 1.30 pm for 1st and 2nd grade of elementary school, and 3.30 pm for 3rd-6th graders. Most children had other extracurricular activities e.g. piano class, math class, swimming class. These arrangements resulted in children arriving home at 6 pm or even 6.30 pm, when they usually had dinner, watched TV or played with their tablets and then went to sleep.

#### Heredity

Carers from low and intermediate SES groups had a similar understanding of heredity i.e. overweight is simply what has been going on in their family for several generations. Carers from high SES groups elaborated further on the concept of heredity based on their personal observation of their children/nephews. A few (5/27) from high SES groups thought that heredity is *not* a factor that contributes to overweight.

### Awareness and feelings towards overweight in children

Relevant excerpts for this category are available in Table 6.

**Table 6:** Quotes on awareness and feelings towards overweight in children according to carers from Bandung, Indonesia

	Low SES	Intermediate SES	High SES
Characteristics of overweight	• P5: If it’s in babies, Ma’am, from here (pointing at her arms), we can see it from their arms. So, in Sundanese, gegeretan (Sundanese term- babies with fat folds; it looks as if their flesh have been sliced). • P7: The size should be the plus one; otherwise it must be sewn. Hahaha • P3: ... for example after they walk for a little while, they go hah hah hah (laboured breathing noises). • P5: Sundanese people said that the way they walk is ngajegag (walking with the leg open, like frog legs), right?	• P8: Uh... the belly is bloated and it looks like the person is pregnant. • P2: When you bring them out to walk, they get tired easily. • P6: They run for a little bit and they are like (makes panting noises). • P7: They are slow; they walk slowly. • P5: So, for example, when the baby is 4 months old, the other child can lie flat on the stomach, but the fat baby can’t do it.	• P4: For example, babies his age are supposed to be in the standing and started to walking stage (but the fat baby cannot do that just yet). P6: Yeah, it’s like, the children seem to experience difficulties to move. It looks difficult for them to walk. I see that a lot, and I use to think, “That is overweight”. • P5: It’s probably because she has tough bones, bigger bones and however much weight she loses she will never be as thin as other kids. The problem is, currently she moves slower than her younger sibling and she has breathing problems, too • P7: If the person is overweight, he’s prone to diseases and becomes sick easily.
Food portion size in overweight children	• P9: So when I gave her the food, I gave her less, I don’t follow the serving size guidelines from the box. • P1: For normal children, it’s just the standard serving...for the overweight children, they will have as much as adults.	• P3: My son (elementary school) eats the same portion as mine. • P7: Like earlier this morning, before I came here, we have breakfast with fried rice. Well, after fried rice, he asked more, he asked for noodles. I gave him one pack of noodle, and he ate it until there’s nothing left. • P4: In a day? Well, actually, he eats three times a day, but the portion is more than me. For me, for example, I eat one full plate. My child can eat up to three full portion.	• P6: She only eats zuppa soup as main meal.P2: How many bowls?P6: Only three bowls. • P5: Because we think the children are still on the growth period (so we let the children eat a lot). • P7: Parents are also afraid if the children get sick, because there are so many diseases right now. • P6: So sometimes they make their children eat a lot to prevent them from getting sick.
Eating frequency in overweight children	• P4: My niece, 5th grade, 58 kg, she eats at 6 pm, then she eats again at 8 pm. Mostly, she eats meatballs, she rarely eats any vegetables. • P2: A child should eat three meals a day right? This one eats more than three times and she eats noodles, sometimes two packages aren’t enough for her, only four is. • P9: My (obese) child eats regularly, three times at home, but even when he has eaten and our neighbour offers him something to eat, he will eat it. When he comes to his aunt’s house and she offers him food, he will eat it.	• P7: He’s always eating. He never stops eating. • P5: He eats four times a day, and he also eats snacks like bread, cakes, sweets. • P2: He eats…he eats at noon. The others only eat in the morning and after Isya (the last Moslem’s prayer time in a day, in Indonesia, it’s at about seven o’clock p.m). When the others eat after Isya, my son also joins them. After that, he watches TV until 11 o’clock. Sometimes, at ten o’clock, he eats again. That means he doesn’t make a move (after his last meal), then he directly goes to sleep.	• P5: Yeah, people do that you know. My son, my eldest one weighs almost 70 k. He eats one meal at 10 am, and then another one at 12. In the afternoon, when he finally gets home, he eats another meal. He just wanders around the kitchen looking for food, and suddenly he has a plate full of rice. • P4: For my child, if he eats regularly like three times a day, his weight is stable. Three meals: morning, noon, and afternoon around six pm. Stable, but once there are snacks. Oh, his weight goes skyrocketing.
Feelings about overweight children	• P5: Who doesn’t want a fat child? • P7: They are cute. I want to pinch them. • P2: I feel gereget (kind of loving something cute mixed with desire to pinch, in Sundanese), Ma’am. • P8: Happy to see a fat child, but pity him as well • P6: Because it’s a pity. It’s difficult. Yes, the fat children have difficulties to move. They become uh, they’re lazy, they sleep a lot. • P7: Out of fear of becoming like A (name), so we limit our child’s diet... • P9: Horrified.	• P7: When they’re little, it’s kind of cute (to be fat). • P3: Yes, cute, like on TV. • P6: It scares me. • P1: If the child is too fat, sometimes I feel pity for him/her, just like the one on TV. • P8: I feel sorry for him, Doctor.	• P8: When we look at the fat children, we’ll think they are cute…But actually it’s a pity, fat children are having difficulties to move. • P6: I’m afraid my child will become that fat. • P2: The problem is that they are vulnerable to diseases, I’m afraid of that. Underweight children are vulnerable to diseases, but so are the big ones, hahaha.
Sensitivity in carers of own overweight children	• P6: This doesn’t happen to my child, he is fat but healthy. • P9: Well, when he runs around, he doesn’t get tired easily.	• P8: I don’t think a fat person gets tired easily because mine is fat (and he does not get tired easily).	• P5: My child, who is fat, is actually very active and can run quickly. • P8: When he’s racing with his thinner friends, he runs faster. • P4: Fat people are not always slower.
Children’s current weight and height	• P2: I just want it [my child’s weight] to be just normal, ascending in a straight line [on the KMS- Kartu Menuju Sehat (Health Card)].	• P3: I have too many under 5 children, everytime I go to Posyandu (community health post) to weigh them; I bring 3 or 4 children. • P4: I thought that my child was the only short one, but it turns out that there are a lot of kids who are as short as he is.	• P7: Importantly, it must be according to the chart, or the doctor’s opinion. If the doctor says okay, I’ll be thankful. • P6: I’m aware that I am short, so my children won’t be tall.

#### Characteristics of overweight

All SES groups mentioned similar characteristics: physical appearance (“bulging belly”, head circumference, “stomach folds”, plump cheeks, “hulk like”, “big butt”, no neck), size of their clothes, difficulty in movements, tiring easily, panting breath, and having a lower IQ level. Intermediate and high SES carers added that overweight children get sick more often compared to their healthy weight counterparts.

#### Food portions in overweight children

All carers stated that overweight children eat bigger portions compared to healthy weight children, admitting that sometimes their children eat the same portion of food as their parents. One mother (low SES group) stated that as one of her efforts to reduce her child’s weight, she gave her smaller portions, different from the recommended serving size on the box of instant food.

#### Eating frequency in overweight children

Carers from the low SES groups were aware that their overweight children eat more than three times a day. One carer stated that her obese child eats regularly three times a day at home, but every time he visits a relative or a neighbour, he accepts their invitation to eat. It is customary in Indonesia to offer people (children or adults) food whenever they visit. It is considered polite and respectful for the host to do so, and the guest should accept, in order to show respect.

Carers in the intermediate and high SES groups also raised the issue of snacks, as their children would eat three or four times a day and snack in between.

#### Feelings about overweight children

Carers from low SES groups had mixed feelings about overweight babies/children, one fourth (9/35) having positive feelings such as happiness and an eagerness to pinch the child to express their feelings. Pinching children on their cheeks to express their feelings is common in Indonesia, for example when people think the child is cute. However, more carers (26/35) had negative feelings such as fear that their children will become overweight, feeling sorry for the overweight child because they might be bullied at school or because of their inability to participate in some of the schools’ physical activities, or feeling that overweight children are “just lazy”.

Similar to carers from the low SES groups, carers from the intermediate SES groups also had mixed feelings about overweight babies/children. Several (6/32) expressed positive feelings but most (26/32) portrayed negative feelings.

All carers from the high SES groups shared the same negative feelings towards overweight children. Carers from all SES groups shared their concern about overweight-related health problems such as heart disease, diabetes, difficulty in breathing, cholesterol problems, or high blood pressure.

#### Sensitivity in carers of overweight children

All carers who mentioned that their children were overweight expressed similar sensitive attitudes when it came to their own children, irrespective of SES. For example, they placed more attention on their children’s capabilities in physical activities rather than their weight status.

#### Children’s current weight and height

Most carers of the low and intermediate SES groups knew their children’s current weight (29/35 and 25/32, respectively), but only some (21/35 and 15/32, respectively) knew their children’s height. These families had their children’s height and weight measured regularly at the *Posyandu* until they were 5 years old. From the carers’ facial expressions and the way they stated their experience of going to *Posyandu* to have the child measured, it is indicative that carers from low and intermediate SES groups may consider this as just an obligation to tick the checklist instead of an opportunity to monitor their children’s development. In public elementary schools, weight and height are measured once a year.

All high SES group carers agreed that their indicator of health is their children’s weight, with 22/27 knowing their children’s current weight, while only a little more than half (15/27) knew their height. Five carers (5/13) admitted they were only concerned about the children’s weight and not height; one even stated she never paid attention to her children’s heights. One mother stated that the only opinion that matters for her was her trusted doctor’s. Under 5 children are weighed during visits to the paediatrician whereas 7–12 year old children are weighed every 2 months at (private) school. Even with this scheme, many carers did not pay much attention to their children’s current weight or height.

One third (35/94) of all carers from all SES groups were satisfied with their children’s growth; (including all carers who stated that their children were healthy weight/overweight), while 59/94 carers were not satisfied. Carers admitted that they compared their children’s weight or height to that of their peers at school, in the playground or other public places. One mother from the intermediate SES groups stated that she compared her son’s height with his friends because she thinks that he is short and felt relieved when she saw that his peers were mostly ‘as short as he is’.

## Discussion

This is the first study in Indonesia that investigates the knowledge, attitudes and beliefs surrounding childhood overweight of mothers/grandmothers from different SES groups. We identified 3 categories and 13 subcategories as stated in Table 3. However, due to word limitations, we can only discuss several subcategories: normal weight range, ideal body shape, characteristics of overweight, activity levels and sedentary behaviour, heredity, how carers define overweight, children’s current weight and height, and chubbier is healthier.

All three SES groups had similar expectations about an appropriate weight at birth, 6 and 12 months. This might have resulted from the participation of mothers in the *Posyandu*, [21] community counselling usually done in *Puskesmas,* or consultations with health practitioner or paediatricians. All participants had similar ideas about the ideal adult body type, demonstrating the influence of the media in their lives. It is very likely children of parent participants watched television with their parents and were influenced by what is considered the ideal adult body type. Thus, children who absorbed large amounts of screen time could relate to the ideal for adults but not have a connection with the young child’s bodyweight because there is no such ideal for children either in the media or in everyday reality. Linking this to inadequate PE in school curricula seems to suggest that a child’s ideal body type is not considered as important. All carers had similar opinions about the characteristics of overweight children: overweight children had bigger portion sizes and ate more frequently compared to their healthy weight counterparts. How carers characterised overweight is similar to studies in the United Kingdom [22] and the United States [23] where parents were found to rely on visual appearance, exercise capability in relation to peers, and clothing sizes.

The carers recognised that dietary factors, activity levels and sedentary behaviours, and heredity all contributed to the development of overweight. Most carers agreed that the 2-h physical education (PE) class at school is not enough, especially as this includes changing clothes and getting to the sportsfield. One qualitative study with US school principals, PE teachers and students highlighted the need for improvement in the in-school physical activity; one student referred to PE class as a “dumping ground”, where children were sent when other “subject matter” teachers were not available [24]. Another US study, using pedometers to count steps, showed that typical PE classes only account for 8–11% of the total steps made throughout the day [25]. A 3-year intervention study of primary school children in Sweden showed that to successfully reduce weight gain, children needed to be physically active for more than 40 min in each physical education setting every day [26]. Carers seemed less worried about physical education at school because children from low and intermediate SES groups spend most of their after school hours playing outside the house, which add to their physical activity hours; while the high SES children engaged more in extracurricular activities.

Many carers assigned heredity or genetic predisposition as the ‘expected and accepted’ cause of childhood overweight, a finding also documented in Australia [27] and the United States [23, 28, 29].

Most carers knew the current weight of their children but less than 60% of these carers knew about their children’s height. Most carers defined overweight subjectively from physical appearance. This finding is in keeping with studies from Germany, [30] the United Kingdom, [22] the United States, [23, 31] and in Vietnam [32]. While some carers knew how to define overweight objectively, this knowledge is not very useful when they do not know their child’s current weight or height. Knowing how to measure overweight and then knowing a child’s weight and height status may be important first steps in recognising whether children are underweight, of a healthy weight or overweight [22, 31–33].

Some carers agreed with the ‘chubbier is healthier’ concept that is also found in other cultures. For example in Turkey (plump kids are healthy), [34] Vietnam (chubbiness is good), [32] the United States (“baby fat” is cute and healthy [31]; a bigger infant is a better infant [28]) and the United Kingdom (a big baby is a healthy baby) [35]. The concept of overweight symbolising family wellbeing is also found in China [36].

Carers from the high SES groups expressed their opinions freely and in a more confident manner compared to the other two groups. This is probably related to their educational background, with more carers from the high SES groups being university graduates, compared to none from the other groups. Within the low SES groups, we found that the women from villages with an active midwife (in terms of educating the carers) were involved more actively in the discussion. This may highlight the significant role played by front line staff in the health system, e.g. midwives and general practitioners. Two studies in Indonesia support this issue, stating that the availability and higher participation in *Posyandu* in Indonesia have significantly improved children’s nutrition status and had a protective effect on the development of overweight in children [21, 37].

One aspect to highlight in our study is the interaction between participants within each focus group, an advantage of conducting FGDs [38]. Some carers supported each other and some questioned another’s opinion, bringing many insights to the topic and richness to our data. Comparison between the three SES groups revealed more similarities in low and intermediate SES groups in the way they perceived childhood overweight and related topics. We also found that in a few subcategories (how carers define overweight; chubbier is healthier), carers from low SES groups shared similar knowledge with their counterparts from the higher SES groups. For some issues, the low SES carers even showed better knowledge compared to their counterparts from higher SES, for example in the knowledge to define overweight objectively and knowledge about the current weight and height of their children.

A strength of this study is that it is the first in Indonesia to investigate the perceptions of overweight in carers from three different SES groups. Secondly, in contrast to the other Indonesian study where only mothers of overweight children were involved, our study recruited carers with children of diverse weight status, providing a variety of perspectives. Thirdly, we had the same number of groups in each status category, allowing us to compare the results between the three groups involved. Fourthly, we successfully attracted participation from carers of low SES groups, even though low SES participants are considered by some to be difficult to recruit [31]. One of our study’s limitations is that because the number of mother participants outweighed the number of grandmother participants, comparison of these two groups’ perspectives was unavailable. Although our qualitative research findings may lack generalisability, we believe our findings are supportive of findings from other countries while producing new replicable findings that will represent carers’ views if done in other regions of Indonesia.

## Conclusions

This study sought to explore the perceptions of carers towards childhood overweight. Although there were similarities in the way carers from different SES groups perceived overweight, the differences were of great interest. In discussion of health-related issues, there were differences on the perceptions of overweight not only between different SES groups but also within the same SES groups.

### Recommendations

Irrespective of these differences, one of the policy implications around the perception of carers is the importance of educating mothers and other primary carers of children around healthier lifestyles. This study highlights the need for improvement within Indonesia in the dissemination of current policies such as PUGS and the use of KMS, for example through the media (newspaper and television). The use of the KMS card needs to be implemented with all levels of health practitioners, making it the national reference in Indonesia to regularly monitor children’s growth. Consideration should be given to routine anthropometric measurements, currently undertaken in private elementary schools, being implemented in every school, thus providing feedback to parents about their children’s weight and height status. There is room for review and revision of the quality and quantity of physical education class in elementary schools. The Department of Education may need to increase the times for children’s physical activities during school hours, e.g. during recess or before and after school physical activities. Future research should aim to explore the food choices made by carers and the involvement of other people in the household (father, grandparents) in making diet-related decisions.

## Additional file

Additional file 1: Kartu Menuju Sehat (KMS- Health Card) for boys aged 0–24 months. Source: http://www.depkes.go.id/resources/download/info-terkini/Kartu%20Menuju%20Sehat%20KMS.pdf. (PDF 2475 kb)

## Footnotes

1 We included carers of under five and elementary school children because of the practicalities in recruiting these mothers through Community Health Centers, private practices and schools. Carers of 5 and 6-year-olds (kindergarten age) are much harder to recruit because kindergarten is not mandatory in Indonesia.

## References

[1] International Food Policy Research Institute (2015). Global nutrition report 2015: actions and accountability to advance nutrition and sustainable development.

[2] Rachmi CN, Li M, Baur L. Overweight and obesity in Indonesia: prevalence and risk factors–a literature review. Public Health. 2017; 10.1016/j.puhe.2017.02.002 28404492

[3] Shrimpton R, Rokx C (2013). The double burden of malnutrition in Indonesia.

[4] Rizona F, Susetyowati S, Lusmilasari L (2016). Mother's feeding behaviours on overweight children. Int J Community Med Public Health.

[5] Tucker P, Irwin JD, He M, Bouck LM, Pollett G (2006). Preschoolers’ dietary behaviours: parents’ perspectives. Can J Diet Pract Res.

[6] Towns N, D'Auria J (2009). Parental perceptions of their child's overweight: an integrative review of the literature. J Pediatr Nurs.

[7] Scaglioni S, Salvioni M, Galimberti C (2008). Influence of parental attitudes in the development of children eating behaviour. Br J Nutr.

[8] Oude Luttikhuis H, Baur L, Jansen H, Shrewsbury VA, O'Malley C, Stolk RP, Summerbell CD. Interventions for treating obesity in children. Cochrane Database Syst Rev. 2009. doi:10.1002/14651858.CD001872.pub2. 10.1002/14651858.CD001872.pub2 19160202

[9] Golley RK, Hendrie GA, Slater A, Corsini N (2011). Interventions that involve parents to improve children's weight-related nutrition intake and activity patterns - what nutrition and activity targets and behaviour change techniques are associated with intervention effectiveness?. Obes Rev.

[10] Niemeier BS, Hektner JM, Enger KB (2012). Parent participation in weight-related health interventions for children and adolescents: a systematic review and meta-analysis. Prev Med.

[11] Gibson EL, Kreichauf S, Wildgruber A, Vogele C, Summerbell CD, Nixon C, Moore H, Douthwaite W, Manios Y, ToyBox-Study G (2012). A narrative review of psychological and educational strategies applied to young children's eating behaviours aimed at reducing obesity risk. Obes Rev.

[12] Indeks Pembangunan Manusia [https://jabar.bps.go.id/Subjek/view/id/26-subjekViewTab3|accordion-daftar-subjek1].

[13] Operational Working Group Ministry of Health: Pedoman Umum Pengelolaan Posyandu. (Health Mo ed. pp. 6–7. Jakarta: Departemen Kesehatan Republik Indonesia; 2011:6–7.

[14] Strauss A (1987). Qualitative analysis for social scientists.

[15] Strauss A, Corbin J (1990). Basics of qualitative research: grounded theory procedures and techniques.

[16] Glaser B, Strauss A (1967). The discovery of grounded theory: strategies for qualitative research.

[17] NVivo qualitative data analysis Software. vol. Version 11: QSR International Pty Ltd.; 2014. http://www.qsrinternational.com/support/faqs/how-do-i-cite-nvivo-10-nvivo-9-or-nvivo-8-in-my-wo.

[18] Kartu Menuju Sehat [http://www.depkes.go.id/resources/download/info-terkini/Kartu Menuju Sehat KMS.pdf].

[19] Inilah Perbedaan ‘4 Sehat 5 Sempurna’ Dengan ‘Gizi Seimbang’ [http://www.depkes.go.id/article/view/16051300001/inilah-perbedaan-4-sehat-5-sempurna-dengan-gizi-seimbang-.html].

[20] Departemen Kesehatan RI. Direktorat Jenderal Bina Kesehatan Masyarakat DKR: Pedoman Umum Gizi Seimbang (PUGS) - panduan untuk petugas. Indonesia DKR ed. Jakarta, Indonesia: Depkes RI; 2003.

[21] Anwar F, Khomsan A, Sukandar D, Riyadi H, Mudjajanto ES (2010). High participation in the Posyandu nutrition program improved children nutritional status. Nutr Res Pract.

[22] Jones AR, Parkinson KN, Drewett RF, Hyland RM, Pearce MS, Adamson AJ (2011). Gateshead millennium study Core T: parental perceptions of weight status in children: the Gateshead millennium study. Int J Obes.

[23] Goodell LS, Pierce MB, Bravo CM, Ferris AM (2008). Parental perceptions of overweight during early childhood. Qual Health Res.

[24] Gamble A, Chatfield SL, Cormack ML, Hallam JS (2017). Not enough time in the day: a qualitative assessment of in-school physical activity policy as viewed by administrators, teachers, and students. J Sch Health.

[25] Tudor-Locke C, Lee SM, Morgan CF, Beighle A, Pangrazi RP (2006). Children's pedometer-determined physical activity during the segmented school day. Med Sci Sports Exerc.

[26] Sollerhed AC, Ejlertsson G (2008). Physical benefits of expanded physical education in primary school: findings from a 3-year intervention study in Sweden. Scand J Med Sci Sports.

[27] Jackson D, McDonald G, Mannix J, Faga P, Firtko A (2005). Mothers’ perceptions of overweight and obesity in their children. Aust J Adv Nurs.

[28] Baughcum AE, Burklow KA, Deeks CM, Powers SW, Whitaker RC (1998). Maternal feeding practices and childhood obesity: a focus group study of low-income mothers. Arch Pediatr Adolesc Med.

[29] Jain A, Sherman SN, Chamberlin LA, Carter Y, Powers SW, Whitaker RC (2001). Why don't low-income mothers worry about their preschoolers being overweight?. Pediatrics.

[30] Sikorski C, Riedel C, Luppa M, Schulze B, Werner P, Konig HH, Riedel-Heller SG (2012). Perception of overweight and obesity from different angles: a qualitative study. Scand J Public Health.

[31] Eli K, Howell K, Fisher PA, Nowicka P (2014). “a little on the heavy side”: a qualitative analysis of parents’ and grandparents’ perceptions of preschoolers’ body weights. BMJ Open.

[32] Do LM, Larsson V, Tran TK, Nguyen HT, Eriksson B, Ascher H (2016). Vietnamese mother's conceptions of childhood overweight: findings from a qualitative study. Glob Health Action.

[33] He M, Evans A (2007). Are parents aware that their children are overweight or obese? Do they care?. Can Fam Physician.

[34] Esenay FI, Yigit R, Erdogan S (2010). Turkish Mothers’ perceptions of their Children’s weight. J Spec Pediatric Nurs.

[35] Southwell O, Fox JR (2011). Maternal perceptions of overweight and obesity in children: a grounded theory study. Br J Health Psychol.

[36] Li J, Lei J, Wen S, Zhou L (2014). Sex disparity and perception of obesity/overweight by parents and grandparents. Paediatr Child Health.

[37] Andriani H, Liao CY, Kuo HW. Association of Maternal and Child Health Center (Posyandu) availability with child weight status in Indonesia: a National Study. Int J Environ Res Public Health. 2016;13 10.3390/ijerph13030293 PMC4808956 26959047

[38] Green J, Thorogood N (2009). Qualitative methods for Health Research. Second edition edn.

