# Supplementary material for: Perceptions of overweight by primary carers (mothers/grandmothers) of under five and elementary school-aged children in Bandung, Indonesia: a qualitative study
Source: Int J Behav Nutr Phys Act. 2017 Jul 27;14:101. doi: 10.1186/s12966-017-0556-1 (PMC5531021; doi:10.1186/s12966-017-0556-1)
Supplement: Additional file 1: — Kartu Menuju Sehat (KMS- Health Card) for boys aged 0–24 months. Source: http://www.depkes.go.id/resources/download/info-terkini/Kartu%20Menuju%20Sehat%20KMS.pdf. (PDF 2475 kb) [file 12966_2017_556_MOESM1_ESM.pdf]

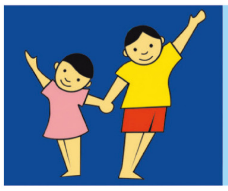

KMS
KARTU MENUJU SEHAT

Untuk Laki-Laki

Nama Anak : ....

Nama Posyandu : ....

Timbanglah Anak Anda Setiap Bulan
Anak Sehat, Tambah Umur, Tambah Berat, Tambah Pandai

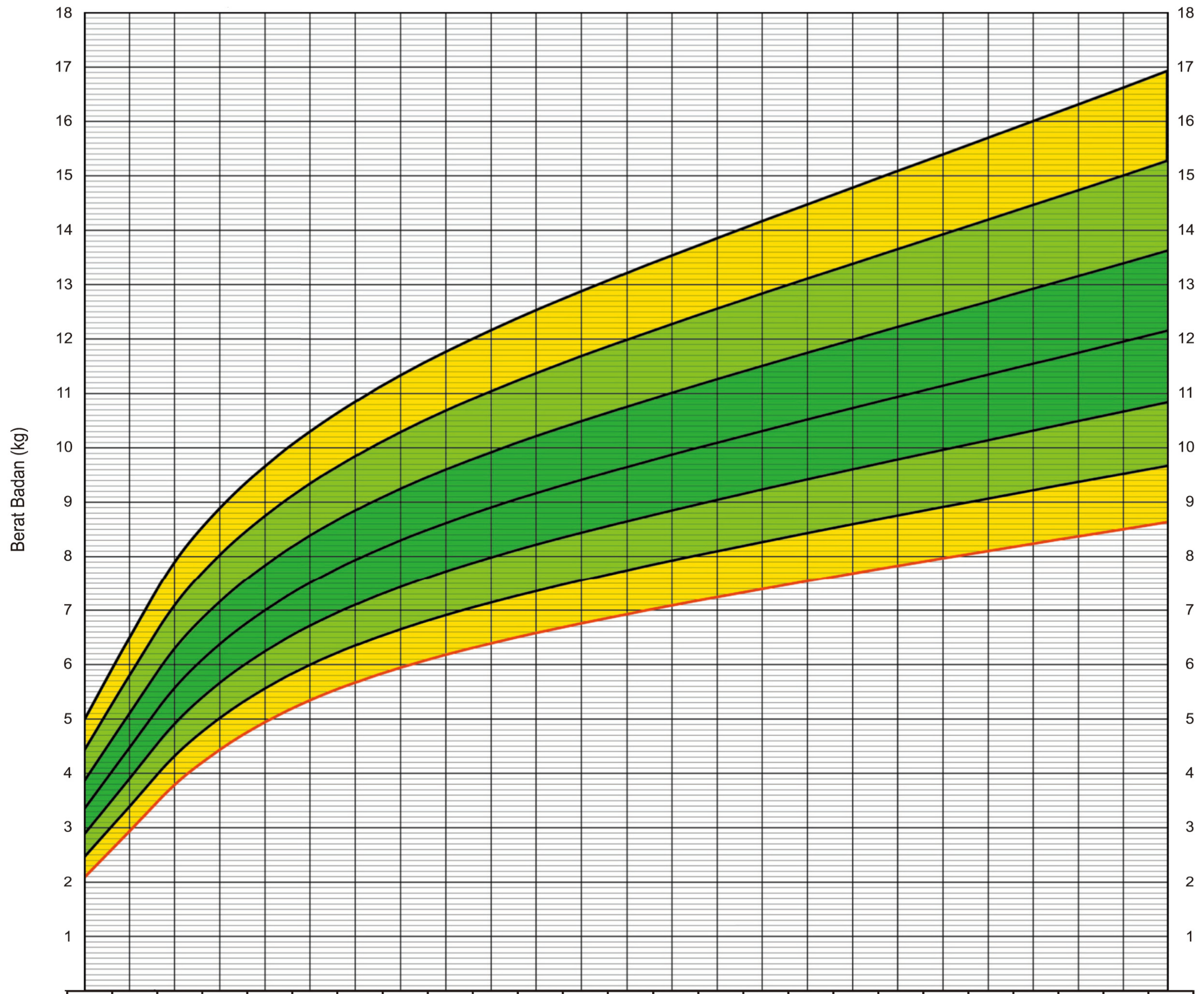

**Umur (bln)**	0	1	2	3	4	5	6	7	8	9	10	11	12	13	14	15	16	17	18	19	20	21	22	23	24
**Bulan penimbangan**																									
**BB (kg)**																									
**KBM (gr)**		800	900	800	600	500	400		300				200												
**N/T**																									
**ASI Eksklusif**																									

NAIK (N)
Grafik BB mengikuti garis pertumbuhan<br>atau<br>Kenaikan BB sama dengan KBM<br>(Kenaikan BB Minimal) atau lebih

TIDAK NAIK (T)
Grafik BB mendatar atau menurun<br>memotong garis pertumbuhan dibawahnya<br>atau<br>Kenaikan BB kurang dari KBM

Rujuk ke petugas kesehatan bila tidak naik 2 kali berturut - turut atau BGM

- *Tanyakan ada tidak kontak dengan penderita TBC (* ya / tidak)*
